# Tunneling Performance Increases at Lower Temperatures for *Solenopsis invicta* (Buren) but not for *Nylanderia fulva* (Mayr)

**DOI:** 10.3390/insects6030686

**Published:** 2015-07-23

**Authors:** Michael T. Bentley, Faith M. Oi, Salvador A. Gezan, Daniel A. Hahn

**Affiliations:** Department of Entomology and Nematology, University of Florida, P.O. Box 110620, Gainesville, FL 32611, USA; E-Mails: Foi@ufl.edu (F.M.O.); sgezan@ufl.edu (S.A.G.); dahahn@ufl.edu (D.A.H.)

**Keywords:** *Nylanderia fulva*, *Solenopsis invicta*, tunneling, temperature, thermoregulation

## Abstract

*Nylanderia fulva* (Mayr), the tawny crazy ant, is an invasive pest established in Florida and several other Gulf Coast states. In their invasive ranges in the Southeastern USA, large *N. fulva* populations have reduced species abundance, even displacing another invasive ant, *Solenopsis invicta* (Buren). In North Florida, *N. fulva* populations survive winter temperatures that reach below freezing for extended periods. However, the shallow littoral debris used by *N. fulva* for nest construction offers little insulation to brood and reproductives when exposed to freezing temperatures. Field populations of *N. fulva* in North Florida were observed tunneling below ground, a previously undescribed behavior. Other invasive ants exhibit similar subterranean tunneling behavior as a means of thermoregulation. To test the hypothesis that *N. fulva* has the capacity to construct subterranean tunnels across a range of ecologically relevant temperatures, tunneling performance for *N. fulva* and *S. invicta*, another invasive ant that tunnels extensively, were compared at four temperatures (15.0, 18.0, 20.0, and 22.0 °C). Overall, *N. fulva* tunneled significantly less than *S. invicta*. *Nylanderia fulva* tunneled furthest at warmer temperatures whereas *S. invicta* tunneled furthest at cooler temperatures. However, *N. fulva* constructed subterranean tunnels at all temperatures evaluated. These data support the hypothesis that *N. fulva* is capable of tunneling in temperatures as low as 15.0 °C, confirming that this ant can also perform a behavior that is used by other ants for cold avoidance.

## 1. Introduction

Biological invasions by non-native organisms can dramatically change the composition and ecology of landscapes [1]. Invasive ants are among the most damaging biological invaders because they can displace native species thus altering the function of entire ecosystems [2]. When moving into a new range, invasive ants may be challenged by temperatures well above or below those in their native range. Subterranean tunneling is one thermoregulatory strategy used to overcome temperature extremes that is exhibited by many invasive ant species including *Solenopsis invicta* (Buren).

*Solenopsis invicta*, the red imported fire ant, is an economically important pest native to South America that is established throughout the Southeastern United States. This mound-building ant species constructs elaborate subterranean nests with tunnel systems that can extend horizontally up to 84 m as well as reach 1.5 m vertically below ground [3,4,5]. Horizontal subterranean tunnels provide *S. invicta* ready access to its foraging territory, even during poor climactic conditions, such as rain or unfavorable temperatures [3]. Vertical subterranean shafts allow *S. invicta* to exploit the temperature-buffering properties of the soil, thus providing a nesting site that maintains relatively stable moisture and temperature conditions during seasonal or sudden weather changes [4,5].

Recently, another South American ant has invaded the Southeastern United States, *Nylanderia fulva* (Mayr). Unlike *S. invicta*’s mound-based colony structure, *N. fulva* generate large polygynous and polydomous colonies commonly located in shallow littoral debris [6]. In rural and urban areas of Columbia where *N. fulva* are established as invaders, foraging populations have reportedly displaced native ant species and injured livestock [6]. In the Southeastern U.S., *N. fulva* colonies have displaced ant species and other arthropods, as well as damaged electrical equipment, landscape goods, and commercial honey bee hives [7,8,9,10].

In Florida, *N. fulva* has been documented to occur in 26 counties throughout the state. While southern Florida’s seasonal temperatures are generally mild, Northern and Central Florida can experience seasonal cold, where overnight air temperatures drop below freezing. To avoid exposure to freezing air temperatures, ant species, such as *S. invicta*, rely on subterranean tunneling as a cold avoidance strategy [4]. However, the ways in which *N. fulva* may avoid cold stress are poorly understood. Existing literature indicates that *N. fulva* rely predominately on shallow littoral debris for protection and nest construction in native and non-native ranges [6]. However, shallow littoral debris typically does not offer much insulation from ambient air temperatures, and thus brood and reproductives housed in littoral nests may be exposed to dangerously cold temperatures during North Florida winters.

Field observations in North Florida suggest that *N. fulva* can tunnel below ground. It is known that subterranean tunneling is part of a thermoregulatory strategy for other ant species, particularly the successful invader *S. invicta*. Therefore, we hypothesize that subterranean tunneling may similarly help *N. fulva* to ameliorate cold stress by buffering colony members from thermal extremes. There are currently no published data regarding *N. fulva*’s tunneling performance or the potential impact of temperature on *N. fulva*’s tunneling behavior. Thus, our objective was to evaluate the extent to which temperature affected *N. fulva*’s tunneling performance, and compare these thermal effects on tunneling to those in *S. invicta*, an invasive ant that tunnels extensively.

## 2. Experimental Section

### 2.1. Collection of Field Colonies

*Nylanderia fulva* were field collected from April to November 2013 as needed, from Alachua and Duval Counties, FL. Debris containing ants and brood were transported to the laboratory in plastic trays (43.0 × 13.0 × 56.0 cm) coated with Fluon^®^ (Insect-A-Slip, BioQuip, Rancho Dominguez, CA, USA) to prevent ant escape. Reference samples of male alates, queens, and workers are maintained at the Florida Department of Agriculture and Consumer Service Division of Plant Industry, Gainesville Florida. Polygynous *S. invicta* colonies were collected from Alachua County, FL, by transferring mounds containing ants into a plastic bucket (19.0 L) coated with talc powder to prevent ant escape. Ants were extracted from soil and other debris using a modified drip-floatation method as described by Banks *et al.* (1981) [11]. One Styrofoam cup (0.7 L) partially filled with paper towel was turned upside down and placed atop the debris within the bucket. As the water level rose within the bucket, ants and brood moved into the cup. Once the water level was above the debris, the cup containing the colony was placed in a plastic tray as described above.

Colonies were kept in the laboratory for ≤2 months, and were maintained at approximately 55.0% ± 8% RH, 27.0 ± 3.0 °C, and 12:12 h L:D photoperiod. Ants were provided with one or more artificial nests constructed of polystyrene petri dishes (100 × 15 mm for *N. fulva*, and 150 × 15 mm for *S. invicta*) partially filled with Castone^®^ Dental Stone (Dentsply International, York, PA, USA) and moistened with deionized water. Colonies were provided a protein source of live termites, dead crickets, or housefly maggots as available, every other day, as well as *ad libitum* access to a moisture source (deionized water) and a carbohydrate source (20% w/v sucrose solution) via glass test tubes plugged with cotton. Water and sucrose levels were checked daily and replaced as needed.

### 2.2. Two-Dimensional Tunneling Assays and Experimental Design

**Figure 1:**
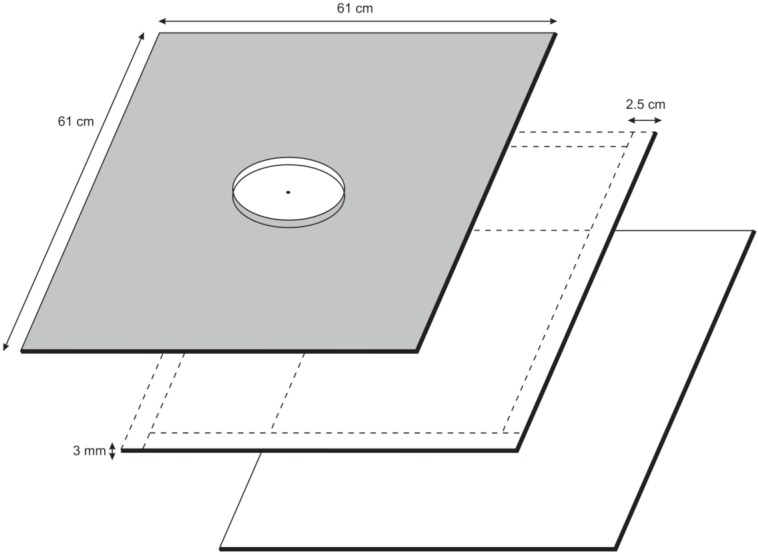
Tunneling arena (61 × 61 cm) constructed of two sheets of transparent Plexiglas (61.0 × 61.0 cm) separated by four Plexiglas strips (61.0 × 2.5 × 0.3 cm) affixed to the outer margins with screws. One access hole (6.5 cm diameter) was drilled at the center of the top sheet and fitted with an ant release chamber.

Ant tunneling performance was evaluated at four temperatures using eight Plexiglas tunneling arenas filled with sand, similar to that described by Puche and Su (2001) [12]. Each arena was constructed of two sheets of transparent Plexiglas (61.0 × 61.0 cm) separated by four Plexiglas strips (61.0 × 2.5 × 0.3 cm) affixed to the outer margins with screws. One access hole (6.5 cm diameter) was drilled at the center of the top sheet and fitted with an ant release chamber (6.5 cm diameter, 177.0 mL, Delta Plastics, Hot Springs, AR, USA) that allowed ants to be introduced into tunneling arenas as well as provided access to deionized water and 20% sucrose solution via test tubes, and live termites (Figure 1).

Ants from four colonies of *N. fulva* and four colonies of *S. invicta* were used. Colony fragments (≈1000 workers, 2 queens, ≈1 mL of brood) from a single colony of each species were assigned in pairs to temperature-controlled chambers in a split plot design with temperature as the whole plot factor and species as the sub-plot factor for a total of 32 experimental units (4 colonies × 4 temperatures × 2 species). Colony fragments paired within one chamber were considered one chamber-set. In total, four seven-day trials were conducted.

Before each trial, colony fragments were held with 20% sucrose solution at 15.0, 18.0, 20.0, or 22.0 °C for 24 h to equilibrate. This temperature range was selected based upon previous studies indicating *S. invicta* and *N. fulva*’s lower thresholds for activity to be approximately 15.0 °C, with increased activity observed at temperatures above 21.0 °C [6,13,14]. Colony fragments were then introduced into tunneling arenas and allowed to tunnel for seven days. Tunnels were measured daily, ants were provided termites as a protein source every other day, and test tubes containing deionized water or 20% sucrose solution were replaced as needed.

To record daily tunneling distance, tunnels were traced on the Plexiglas with a dry erase marker (Expo^®^, Sanford Corporation, Atlanta, GA, USA) using a different colored marker each day. Tunneling arenas were placed over a photographic light box to illuminate tunnels, which improved accuracy of tracing. During the measurement process, individual tunneling arenas were removed from incubators for ≤5 min to minimize thermal change. For each trial, daily tunneling distance per species was summed by temperature to obtain cumulative tunneling data per temperature per species.

### 2.3. Analysis

Cumulative tunneling data for each species and temperature were log-transformed to meet normality assumptions, and analyzed using mixed model analysis (Proc Mixed SAS version 9.3, SAS Institute, Cary, NC, USA) with trial, species, and temperature as fixed effects, and colony and chamber-set as random effects. The interaction term species*temperature was significant, therefore we analyzed the relationship of temperature and tunneling distance separately for each species using a two-way ANOVA where temperature was the fixed effect and colony was the blocking factor. Additionally, least significant differences tests (LSD) (α = 0.05) were used to compare treatment means between species. Data in all figures are presented as arithmetic means.

## 3. Results

**Figure 2:**
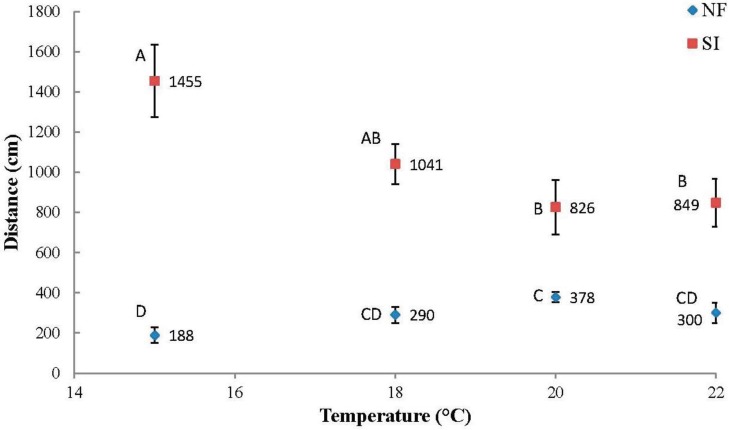
Mean total (±SE) tunneling distances (cm) per temperature for *Nylanderia fulva* (NF) and *Solenopsis invicta* (SI). Mean values within species not sharing the same letter are significantly different (*p* < 0.05, LSD standardized test (SAS version 9.3, SAS Institute, Cary, NC, USA)).

Overall, *N. fulva* tunneled significantly less than *S. invicta* (Figure 2, Table 1). *Nylanderia fulva* tunneled most at higher temperatures, tunneling 101.0% farther at 20.0 °C than at 15.0 °C (Figure 2, Table 2). In contrast, *S. invicta* tunneled most at lower temperatures, tunneling 76.0% further at 15.0 °C than at 20 °C.

**Table 1:** A mixed model analysis evaluating the tunneling performance of *Nylanderia fulva* and *Solenopsis invicta* at 15.0, 18.0, 20.0, and 22.0 °C.

Source	df	Type III F	*p*
Trial	3, 9	2.25	0.1514
Species	1, 12	229.79	<0.0001
Temperature	3, 9	0.44	0.7310
Species*Temperature	3, 12	11.00	0.0009

**Table 2:** A two-way ANOVA summary table by species evaluating the tunneling performance of *Nylanderia fulva* and *Solenopsis invicta* at 15.0, 18.0, 20.0, and 22.0 °C.

Species	Source	df	*F*	*p*
*Nylanderia fulva*	Model	6	3.49	0.0459
	Temperature	3	4.80	0.0290
	Colony (Random)	3	2.17	0.1619
	Error	9		
	Total	15		
*Solenopsis invicta*	Model	6	4.85	0.0177
	Temperature	3	6.55	0.0122
	Colony (Random)	3	3.15	0.0794
	Error	9		
	Total	15		

**Figure 3:**
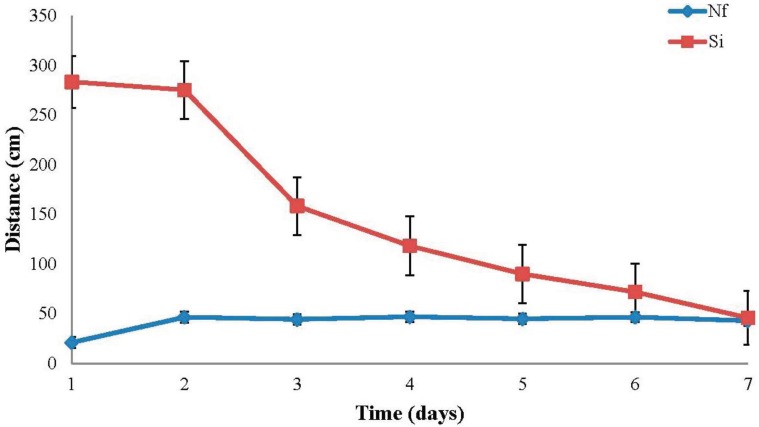
Mean (±SE) daily tunneling distances (cm) for *Nylanderia fulva* (Nf) and *Solenopsis invicta* (Si) over all temperatures.

**Figure 4:**
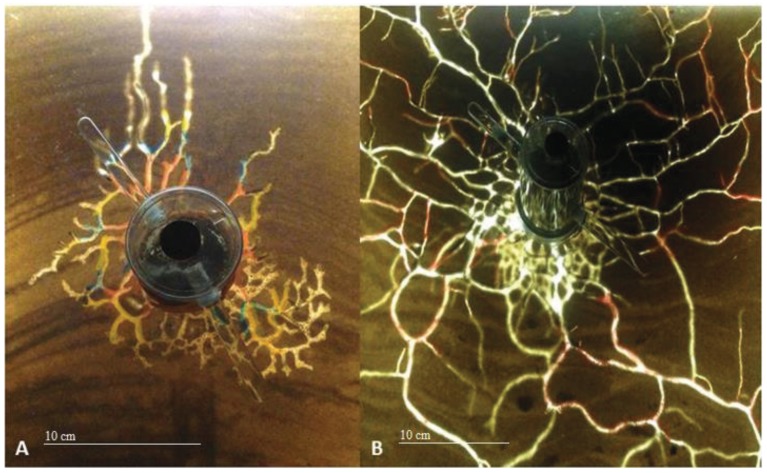
Tunneling performance at 20.0 °C after three days for *Nylanderia fulva* (**A**) and *Solenopsis invicta* (**B**).

*Nylanderia fulva* tunneled an average of 41.7 cm per day over all temperatures. Mean daily tunneling performance for *N. fulva* more than doubled from day one to day two, but remained nearly constant from day two through day seven (Figure 3). *Solenopsis invicta* tunneled an average of 149.0 cm per day overall. Mean daily tunneling performance for *S. invicta* decreased marginally from day one to day two, but decreased by more than 500% from day two to day seven with the greatest drop in tunneling performance occurring between days two and three. Overall, *N. fulva*’s daily tunneling performance was noticeably lower than *S. invicta*’s at all temperatures (Figure 2 and Figure 4).

## 4. Conclusions

To our knowledge, this is the first study to evaluate the effect of temperature on the tunneling performance of *N. fulva* and *S. invicta*. These results support the hypothesis that *N. fulva* has the capacity to construct subterranean tunnels across a range of temperatures that would occur during seasonally cool periods. One study evaluating the thermoregulatory properties of subterranean tunnels demonstrated that tunnels only 10.0 cm deep could be up to 10.0 °C warmer than ambient air temperatures [15]. Similarly, Frouz (2000) [16] reported that temperatures only 3.0 cm below soil surface could be up to 9.8 °C warmer than air temperatures. These results combined with our data suggest that *N. fulva*, like *S. invicta*, could rely on subterranean tunnels to aid in nest thermoregulation during the process of spreading into more temperate ranges. To adequately examine *N. fulva*’s use of subterranean tunneling as a means of thermoregulation, further research is needed to measure *N. fulva*’s capacity for tunneling depth. Furthermore, additional investigation of *N. fulva*’s tunneling performance at upper and lower thermal limits are required.

*Solenopsis invicta*’s strong tunneling performance was consistent with previous studies documenting *S. invicta*’s extensive use of tunneling as a means of foraging, nest construction, and thermoregulation [3,4,5]; however, the negative relationship between temperature and tunneling performance we documented for *S. invicta* contradicts what is often reported in ants [13,17,18,19]. As ectotherms, ants rely on environmental heat to thermoregulate, thus a positive relationship between temperature and activity is typically observed [13,17,18,19]. *Solenopsis invicta*’s greater tunneling performance at lower temperatures may be attributed to its use of subterranean tunneling as a thermoregulatory strategy. Deep subterranean nests allow *S. invicta* to exploit the temperature buffering properties of the soil, allowing ants to shift to regions within the nest with preferred temperatures during seasonal or sudden daily changes [13,18]. *S. invicta*’s increased tunneling performance at 15.0 °C may have been a behavioral response to minimize exposure to an undesirable temperature. To test this hypothesis, further evaluation of *S. invicta*’s tunneling performance across a thermal gradient is needed.

*Solenopsis invicta*’s mean daily tunneling performance at day one was nearly 1250% greater than that of *N. fulva*. However, by day seven, mean daily tunneling performance was almost equal for both ant species. *Solenopsis invicta*’s considerable decline in daily tunneling performance may have been an effect of the finite amount of available space and sand for ants to excavate within the tunneling arenas. During the first two days of each study, *S. invicta* were observed excavating long, wide tunnels that quickly reached the edges of the arena and occupied most of the available tunneling space. Over the remaining five days, *S. invicta* workers were limited in the amount of unoccupied space to construct new tunnels thus excavating smaller, narrower connecting tunnels (Figure 4). These observations may suggest that *S. invicta*’s decline in daily tunneling activity was a result of the spatial limits of our tunneling arenas and not typical of *S. invicta*’s daily tunneling performance in the field. To adequately evaluate this hypothesis, additional investigation of *S. invicta*’s tunneling performance using larger tunneling arenas would be necessary.

Our data demonstrating *N. fulva*’s tunneling performance at temperatures as low as 15.0 °C may also improve seasonal monitoring and treatment programs for this pest ant. *Nylanderia fulva* populations in North Florida typically reach peak densities from early summer to mid fall, making management of this pest very difficult during this period. One study evaluating *N. fulva*’s seasonal activity in North Florida found that satellite nest sites and foraging activity were greatly reduced throughout seasonal cold months [14]. During periods of seasonal cold (≤15.0 °C) it is also likely that *N. fulva* colonies contract, becoming localized around more permanent nests as seen in other invasive ant species [20]. Management of *N. fulva* could be more effective during periods of seasonal cold when *N. fulva* densities are low, and are centralized in smaller areas where nest sites containing reproductives are present. Currently, management programs for *N. fulva* involve inspection and treatment methods that predominantly target the littoral debris commonly associated with the nesting habits of this species. Our results demonstrating *N. fulva*’s tunneling performance suggests that *N. fulva* can tunnel below littoral debris and possibly even into the soil, potentially sheltering this pest ant from current inspection and treatment methods. Therefore, monitoring and control programs occurring during periods of seasonal cold should incorporate sub-soil inspection and treatment methods to facilitate *N. fulva* nest site detection to improve *N. fulva*’s management. 
